# Influence of low-flow time on survival after extracorporeal cardiopulmonary resuscitation (eCPR)

**DOI:** 10.1186/s13054-017-1744-8

**Published:** 2017-06-22

**Authors:** Tobias Wengenmayer, Stephan Rombach, Florian Ramshorn, Paul Biever, Christoph Bode, Daniel Duerschmied, Dawid L. Staudacher

**Affiliations:** 1 Department of Cardiology and Angiology I, Heart Center, University of Freiburg, Hugstetterstrasse 55, 79106 Freiburg, Germany; 2 Department of Medicine III (Interdisciplinary Medical Intensive Care), Faculty of Medicine, University Medical Center - University of Freiburg, Freiburg, Germany

**Keywords:** Venoarterial extracorporeal membrane oxygenation, ECLS, Cardiac arrest, Outcome, Low flow, Chest compression

## Abstract

**Background:**

Venoarterial extracorporeal membrane oxygenation (VA-ECMO) support under extracorporeal cardiopulmonary resuscitation (eCPR) is the last option and may be offered to selected patients. Several factors predict outcome in these patients, including initial heart rhythm, comorbidities, and bystander cardiopulmonary resuscitation (CPR). We evaluated outcomes of all VA-ECMO patients treated within the last 5 years at our center in respect to low-flow duration during CPR.

**Methods:**

We report retrospective registry data on all patients with eCPR treated at a university hospital between October 2010 and May 2016.

**Results:**

A total of 133 patients (mean age 58.7 ± 2.6 years, Simplified Acute Physiology Score II score at admission 48.1 ± 3.4) were included in the analysis. The indication for eCPR was either in-hospital or out-of-hospital cardiac arrest without return of spontaneous circulation (*n* = 74 and 59, respectively). There was a significant difference in survival rates between groups (eCPR in-hospital cardiac arrest [IHCA] 18.9%, eCPR out-of-hospital cardiac arrest [OHCA] 8.5%; *p* < 0.042). Mean low-flow duration (i.e., duration of mechanical CPR until VA-ECMO support) was 59.6 ± 5.0 minutes in all patients and significantly shorter in IHCA patients than in OHCA patients (49.6 ± 5.9 vs. 72.2 ± 7.4 minutes, *p* = 0.001). Low-flow time strongly correlated with survival (*p* < 0.001) and was an independent predictor of mortality.

**Conclusions:**

Time to full support is an important and alterable predictor of patient survival in eCPR, suggesting that VA-ECMO therapy should be established as fast as possible in the selected patients destined for eCPR.

**Electronic supplementary material:**

The online version of this article (doi:10.1186/s13054-017-1744-8) contains supplementary material, which is available to authorized users.

## Background

Chances for surviving cardiogenic shock [1] or cardiopulmonary resuscitation (CPR) are low [2–4]. Some trials suggest clinical improvement after implanting a mechanical circulatory device in patients with shock or after CPR [4, 5]. For patients under resuscitation and not reaching return of spontaneous circulation (ROSC), implanting a venoarterial extracorporeal membrane oxygenation (VA-ECMO) system (extracorporeal cardiopulmonary resuscitation [eCPR]) is the only remaining option [4].

Because time without sufficient circulation correlates with poor prognosis in patients in cardiogenic shock as well as in patients receiving CPR [1, 2], it appears reasonable that VA-ECMO should be available with the shortest delay possible when indicated in selected individuals. Although eCPR might be lifesaving for selected individuals, implantation and management of the VA-ECMO system remains highly challenging and is associated with complications such as bleeding, thromboembolism, limb ischemia, vasoplegia, and others [6]. In case of early initiation of eCPR, the prognosis of patients with spontaneous ROSC might therefore be reduced by VA-ECMO complications. Furthermore, the socioeconomic resources required for successful eCPR are vast. However, it is almost impossible to predict whether an individual will reach ROSC without VA-ECMO within the next minutes.

Despite the challenges and unanswered questions, the use of VA-ECMO has been growing in the last several years. In Germany, there was a 30-fold increase of VA-ECMO administration from 2007 to 2014 (from 96 to 2873 cases) [7]. The broad availability of VA-ECMO might lead to unselective implants and thus to futile care, as well as waste of valuable resources. Considering that patients requiring VA-ECMO represent a very heterogeneous population with very diverse overall prognoses, reliable predictors of favorable outcome are desperately needed. We evaluated outcome with regard to CPR duration until full VA-ECMO support in an all-comers population treated at the Heart Center of the University of Freiburg – Bad Krozingen to investigate whether time until full support may be predictive of survival.

## Methods

We report retrospective data of a single-center registry of patients on VA-ECMO. All patients presenting at the Heart Center at the University of Freiburg – Bad Krozingen between October 2010 and November May 2016 were registered. Patient identity data derived from the registry were blinded, and the study plan was approved by the local ethics committee (EK-Freiburg 151/14). For data analysis, *t* tests, chi-square tests, and one-way analysis of variance were employed where applicable, and a *p* value ≤0.05 was considered statistically significant. All values are given as mean ± 95% CI if not otherwise stated.

### Patient selection

Between October 2010 and May 2016, a total of 133 VA-ECMO device implants during resuscitation were performed. Survival was defined as discharge from the hospital. In-hospital cardiac arrest (IHCA) was defined as cardiac arrest within a hospital, and out-of-hospital cardiac arrest (OHCA) was defined as cardiac arrest outside a hospital with or without the presence of emergency medical personnel.

### ECMO center

Our institution features a 24/7 extracorporeal membrane oxygenation (ECMO) center localized within a tertiary hospital with a 30-bed medical intensive care unit. The VA-ECMO response team consists of one experienced cardiologist/intensivist and one perfusionist.

### ECMO device implantation and management

Indication for VA-ECMO was at the discretion of the responsible physician of the ECMO response team. Our cannulation policy encourages early alarm of the ECPR team and bedside decision-making. Every potential eCPR patient (whether for IHCA or OHCA) triggered an activation of the ECMO team. The decision whether to cannulate was then made at bedside. No cannulation was performed outside the hospital. Typical reasons for not cannulating were age older than 75 years, significant comorbidities, or nonwitnessed cardiac arrest. Cannulation for VA-ECMO was performed preferably bifemorally using Seldinger’s technique without primary surgical cutdown in all cases. Typical venous (draining) cannulas were either 21 French or 23 French in diameter, whereas arterial (returning) cannulas were either 15 French or 17 French in diameter. For patients without active bleeding, anticoagulation was provided by administering unfractionated heparin, aiming at a partial thromboplastin time of 50–60 seconds. The management of vasopressors and fluid therapy was driven by clinical judgment of the treating ECMO experienced intensivist following local standard operating procedures and has been reported before [8].

## Results

### Patient population

A total of 133 patients treated with VA-ECMO were included in this registry. The mean (SD) age at the time of VA-ECMO device implant was 58.7 ± 2.6 years, and a total of 74.4% of all patients were male. The average Simplified Acute Physiology Score II (SAPS2) score at admission was 48.1 ± 3.4, and a proportion of 14.3% of all patients survived their hospital stay. The average time on VA-ECMO therapy was 49.8 ± 9.1 h among all patients, 70.9 ± 22.8 h among survivors, and 46.5 ± 9.9 h among nonsurvivors (*p* = 0.068). Patients with IHCA were older (65.6 vs. 50.1 years, *p* = 0.001) and had shorter low-flow times (49.6 vs. 72.2 minutes, *p* = 0.001) than patients with OHCA. No-flow duration was 2.6 ± 0.8 minutes in the whole cohort (OHCA 5.4 vs. IHCA 0.3 minutes, *p* = 0.001). Both groups displayed similar 10-item Therapeutic Intervention Scoring System (TISS-10) and SAPS2 scores at admission (Table 1). We observed no statistical differences in no-flow times, initial rhythms, immediate coronary angiography results, or TISS-10 or SAPS2 scores when we compared survivors with nonsurvivors (Additional file 1: Table S1).

**Table 1:** Patient and event characteristics

	All	eCPR OHCA	eCPR IHCA	*p* Value
Number of patients	133	59	74	
Age, years	58.7 ± 2.6	50.1 ± 4.0	65.6 ± 2.7	**0.001**
Female sex	25.6%	18.6%	31.1%	0.102
TISS-10 score at admission	21.5 ± 1.8	22.4 ± 2.1	21.0 ± 2.5	0.462
SAPS2 score at admission	48.1 ± 3.4	46.1 ± 6.8	49.0 ± 3.9	0.428
Low-flow time, minutes	59.6 ± 5.0	72.2 ± 7.4	49.6 ± 5.9	**0.001**
No-flow time, minutes	2.6 ± 0.8	5.4 ± 1.5	0.3 ± 0.3	**0.001**
Preexisting conditions				
CAD	57.1%	49.2%	63.5%	0.119
Arterial hypertension	49.6%	33.9%	62.2%	**0.001**
PAD	10.5%	8.5%	12.0%	0.511
COPD	7.5%	5.1%	9.3%	0.357
Other pulmonary disease	4.5%	1.7%	6.7%	0.170
Liver disease	9.0%	1.7%	14.7%	**0.009**
Kidney disease	27.1%	16.9%	35.1%	**0.022**
Diabetes	27.8%	22.0%	32.4%	0.203

Data are shown as mean ± 95% CI or as percentage of patients

*Abbreviations: CAD* Coronary artery disease, *COPD* Chronic obstructive pulmonary disease, *eCPR* Extracorporeal cardiopulmonary resuscitation, *IHCA* In-hospital cardiac arrest, *OHCA* Out-of-hospital cardiac arrest, *PAD* Peripheral arterial disease, *SAPS2* Simplified Acute Physiology Score II, *TISS-10* 10-item Therapeutic Intervention Scoring System

### Patient survival

**Fig. 1:**
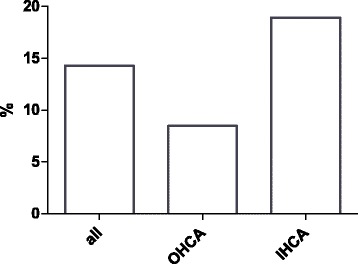
Mean survival of all extracorporeal cardiopulmonary resuscitation patients. *OHCA* Out-of-hospital cardiac arrest, *IHCA* In-hospital cardiac arrest

Survival in all patients after eCPR was 14.3% (Fig. 1). Mean duration between collapse and the beginning of resuscitation (no-flow time) was 2.6 ± 0.8 minutes. There was no difference between survivors and nonsurvivors (*p* = 0.528).

**Fig. 2:**
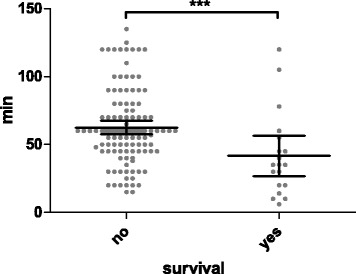
Scatterplot of low-flow time in survivors and nonsurvivors (*** *p* = 0.003). Low-flow time means duration of mechanical cardiopulmonary resuscitation before full extracorporeal membrane oxygenation support

**Fig. 3:**
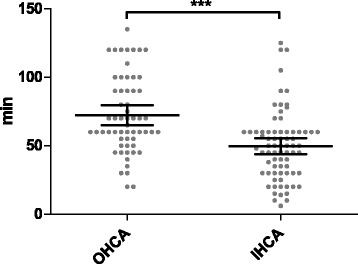
Scatterplot of low-flow time in out-of-hospital cardiac arrest (OHCA) and in-hospital cardiac arrest (IHCA) patients (*** *p* = 0.001). Low-flow time means duration of mechanical cardiopulmonary resuscitation before full extracorporeal membrane oxygenation support

The mean duration between collapse and the beginning of full extracorporeal support was 59.6 ± 5.0 minutes. Patients who survived the hospital stay had significantly shorter low-flow duration than nonsurvivors (41.7 ± 15.0 vs. 62.6 ± 5.1 minutes, *p* = 0.003) (Fig. 2). Prognosis after cardiac arrest was significantly worse in patients after OHCA than after IHCA (8.5% vs. 18.9%, *p* = 0.042) (Fig. 1). OHCA and IHCA patients had significantly longer low-flow times before reaching full VA-ECMO support (72.2 ± 7.4 vs. 49.6 ± 5.9 minutes, *p* = 0.001) (Fig. 3).

**Fig. 4:**
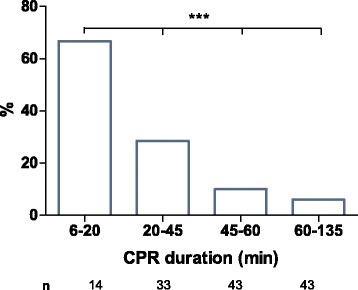
Mean survival for extracorporeal cardiopulmonary resuscitation patients after 6–20, 20–45, 45–60, and 60–135 minutes of mechanical cardiopulmonary resuscitation (CPR) (*** *p* = 0.001)

There was a significant, negative, linear correlation between time to circulatory support and survival in the overall eCPR population (*p* < 0.001, *r* = 0.266) (Fig. 4), indicating that shorter time to circulatory support was associated with better prognosis. This correlation was seen in IHCA patients as well as OHCA patients and persisted after adjustment for baseline characteristics, including age, initial rhythm, and immediate coronary angiography. Survival rates were 67% in patients (*n* = 14) with a CPR duration shorter than 20 minutes and 29% (*n* = 33), 10% (*n* = 43), and 6% (*n* = 43) after 20–45, 45–60, and 60–135 minutes of mechanical CPR, respectively.

**Fig. 5:**
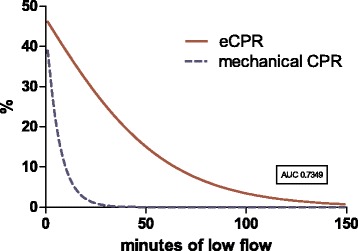
Estimated survival rates for extracorporeal membrane oxygenation (eCPR) patients after every given low-flow time (*red line*). For comparison, data from Goto et al. [16] representing survival after mechanical cardiopulmonary resuscitation (CPR) are included (*dashed blue line*)

We developed a logarithmic probability model for survival based on low-flow time until VA-ECMO support (Fig. 5). Calculated chances of survival were 30%, 20%, 10%, 5%, and 1% at minutes 22, 39, 64, 87, and 139, respectively.

## Discussion

Although it is known that prognosis rapidly decreases during CPR without ROSC, the time point at which prognosis is close to zero in conventional CPR (hence indicating that continued CPR may be futile) remains controversial and has been suggested to range from 16 minutes [2] to 40 minutes [3]. CPR itself is a state of hemodynamic low flow determining the decelerating prognosis over time. Implanting a VA-ECMO device during CPR does not directly address the underlying disease (with the exception of cases of primary hypoxia, hypercapnia, or hypothermia), but it stabilizes the patient until curative treatment can be administered (e.g., by percutaneous coronary intervention in patients with myocardial infarction). VA-ECMO provides the required time to diagnose and treat the variety of underlying conditions, including pulmonary embolism, myocardial infarction, sepsis, intoxication, electrical storm, and others. Furthermore, the state of low flow that ultimately leads to multiorgan failure is averted.

It is conceivable that patients who receive eCPR may have a better prognosis than those who receive conventional CPR. Chen and coworkers demonstrated that patients treated with eCPR had a short-term and long-term survival benefit compared with patients treated with conventional CPR. However, this was a highly selected cohort of 59 patients [9]. In 2011, Shin and colleagues confirmed this finding in a larger propensity score-matched retrospective, single-center, observational study [10].

More recently, other groups have reported eCPR survival rates as high as 15% after OHCA with a surprisingly long average time from collapse to VA-ECMO of 77 ± 51 minutes [11]. The sample size was smaller (*n* = 26 compared with 133), and the included patients were younger (40 compared with 50 years) than in our cohort. eCPR still is a very new technique, ECMO centers around the world are still learning it, and the number of cases is still low.

However, these data, together with ours, suggest that prolonged resuscitation may not be futile if eCPR can be administered [11]. More important, our data show that time from collapse to full support strongly correlates with survival, with best survival with shortest low-flow duration. A recent publication even suggested very little impact of cardiac arrest itself on mortality in ECMO patients as long as sufficient VA-ECMO support was available [12]. Our OHCA patients were significantly younger and had fewer comorbidities than IHCA patients, but they had longer CPR durations and poorer outcomes. This might illustrate the importance of time to full support in eCPR because IHCA patients had shorter low-flow times. The short average no-flow time of 2.6 minutes might be explained by our center policy to rarely cannulate patients with nonwitnessed cardiac arrest.

Chances of survival with favorable neurological outcome after 30 minutes of conventional resuscitation are generally reported to be below 1% [2]. Successful resuscitations with a conventional CPR duration longer than 60 minutes are described in case series reports only [13]. Despite these negative results, many resuscitation teams continue CPR even after 30 minutes. In an observational study including 31,000 patients, CPR duration was longer than 30 minutes in 14% of the cases [14]. Generally, the resuscitation team integrates factors such as initial rhythm, age, comorbidities, no-flow time, and CPR duration, yielding very individualized decisions. Reflecting this, the 2015 European Resuscitation Council Guidelines for Resuscitation state that resuscitation should not be aborted for a single reason [15], such as duration of resuscitation. In our registry, survival strongly correlated with low-flow time (Fig. 2). Using these results, we calculated a survival curve for eCPR patients determined by low-flow duration (Fig. 3). Our model calculation showed that chances of survival were still 25.2% after 30 minutes and 9.9% after 65 minutes of mechanical resuscitation before full VA-ECMO support. This suggests a number needed to treat of 10 with eCPR, including a low-flow time longer than 60 minutes. We included data from Goto et al. [16] in the figure representing 17,238 patients from Japan after OHCA as a reference for survival probability without eCPR for comparison. Because our registry features eCPR patients only and no randomization has been performed, this conclusion cannot be made. A historical control group has significant limitations, as does a comparison with patients at our institution not undergoing eCPR (for selection bias). One therefore must focus on low-flow duration comparing eCPR patients only.

If emergency clinicians, rescue personnel, and ECMO specialists cooperate to further shorten low-flow times and accelerate VA-ECMO system implantation in selected patients without ROSC, outcome after cardiac arrest will likely improve. According to our data, once VA-ECMO is considered a therapeutic option, the delay until arrival of the ECMO team should be minimized.

### Authors opinion on how to improve outcome

The best strategy for delivering VA-ECMO support, however, still needs to be determined. Whether a mobile VA-ECMO team is faster than, and at least equally effective as, a prepared VA-ECMO team within a cardiac arrest receiving team waiting for the patient at the center is still unclear. Our registry includes VA-ECMO cases with ECMO device implantation within the hospital only. Transportation of patients under resuscitation is problematic for several reasons. First, medical personnel are at risk during transportation, and second, quality of CPR in a moving vehicle might be inefficient. Researchers in several trials have investigated mechanical chest compression devices, indicating that CPR with these devices is noninferior to manual CPR [17–19] and might enable early transportation. An accelerated CPR algorithm with ultrarapid transportation with the use of a mechanical chest compression device might be an efficient strategy to minimize time to VA-ECMO support. Awareness of eCPR and early activation of the ECMO team are mandatory for shortening low-flow times and should be implemented in local standard operating procedures.

### Limitations

This is a retrospective observational study of eCPR patients; a selection bias therefore has to be presumed. We report no neurological outcome and no long-term outcome. We included all eCPR patients, regardless of the underlying causes of cardiac arrest. Because it is still not clear which candidates might benefit more from eCPR, we think this approach is reasonable and should be regarded as an all-comers registry.

## Conclusions

eCPR rates of survival after OHCA and IHCA are meaningful and need to be increased further. Our data suggest that for both entities, low-flow duration determines outcomes in eCPR. Low-flow time can and must be minimized. Finding the right point in time to switch from conventional CPR to eCPR remains challenging and cannot be answered with registry data. Robust data comparing a fast-switch (or instant) regime with a conventional regime is desperately needed.

## Additional file

Additional file 1: Patient and event characteristics: survivors vs. nonsurvivors. (DOCX 56 kb)

## Abbreviations

CAD: Coronary artery disease
COPD: Chronic obstructive pulmonary disease
CPR: Cardiopulmonary resuscitation
ECMO: Extracorporeal membrane oxygenation
eCPR: Extracorporeal cardiopulmonary resuscitation
ICU: Intensive care unit
IHCA: In-hospital cardiac arrest
OHCA: Out-of-hospital cardiac arrest
PAD: Peripheral arterial disease
ROSC: Return of spontaneous circulation
SAPS2: Simplified Acute Physiology Score II
TISS-10: 10-item Therapeutic Intervention Scoring System
VA-ECMO: Venoarterial extracorporeal membrane oxygenation
