# Supplementary material for: Influence of low-flow time on survival after extracorporeal cardiopulmonary resuscitation (eCPR)
Source: Crit Care. 2017 Jun 22;21:157. doi: 10.1186/s13054-017-1744-8 (PMC5480193; doi:10.1186/s13054-017-1744-8)
Supplement: Additional file 1: — Patient and event characteristics: survivors vs. nonsurvivors. (DOCX 56 kb) [file 13054_2017_1744_MOESM1_ESM.docx]

**Patient and event characteristics – survivors vs. non-survivors**

|  | **all** | **survivors** | **non-survivors** | p value |
| --- | --- | --- | --- | --- |
| n | 133 | 19 | 114 |  |
| age | 58.7 ± 2.6 | 58.3 ± 9.8 | 58.8 ± 2.7 | 0.889 |
| gender (female) | 25.6% | 52.6% | 21.1% | 0.003 |
|  |  |  |  |  |
| TISS10 at admission | 21.5 ± 1.8 | 19.8 ± 3.1 | 22.0 ± 2.2 | 0.284 |
| SAPS2 at admission | 48.1 ± 3.4 | 43.1 ± 7.9 | 49.7 ± 3.7 | 0.097 |
|  |  |  |  |  |
| Low flow time (min) | 59.6 ± 5.0 | 41.7 ± 15.0 | 62.6 ± 5.1 | 0.003 |
| no flow time (min) | 2.6 ± 0.8 | 2.0 ± 1.8 | 2.7 ± 0.9 | 0.528 |
|  |  |  |  |  |
| **Initial rhythm** |  |  |  |  |
| VT/VF | 38.6% | 50.0% | 36.8% | 0.287 |
| Immediate coronary angiography | 81.1% | 85.7% | 80.6% | 0.642 |
|  |  |  |  |  |
| **Pre-existing conditions** |  |  |  |  |
| CAD | 57.1% | 57.9% | 57.0% | 0.943 |
| Arterial hypertension | 49.6% | 42.1% | 50.9% | 0.479 |
| PAD | 10.5% | 5.3% | 11.4% | 0.419 |
| COPD | 7.5% | 5.3% | 7.9% | 0.687 |
| Other pulmonary disease | 4.5% | 5.3% | 4.4% | 0.865 |
| Liver disease | 9.0% | 21.1% | 7.0% | 0.048 |
| Kidney disease | 27.1% | 31.6% | 26.3% | 0.633 |
| Diabetes | 27.8% | 26.3% | 28.1% | 0.874 |
